# A selective laser-based sensor for fugitive methane emissions

**DOI:** 10.1038/s41598-023-28668-z

**Published:** 2023-01-28

**Authors:** Mhanna Mhanna, Mohamed Sy, Aamir Farooq

**Affiliations:** Mechanical Engineering Program, Physical Science and Engineering Division, Clean Combustion Research Center, King Abdullah University of Science and Technology (KAUST), 23955-6900 Thuwal, Saudi Arabia

**Keywords:** Climate sciences, Environmental sciences, Energy science and technology, Engineering, Optics and photonics, Physics

## Abstract

A mid-infrared laser-based sensor is reported for the quantification of fugitive methane emissions. The sensor is based on a distributed feedback inter-band cascade laser operating near 3.3 μm. Wavelength tuning with cepstral analysis is employed to isolate methane absorbance from (1) fluctuations in the baseline laser intensity, and (2) interfering species. Cepstral analysis creates a modified form of the time-domain molecular free-induction-decay (m-FID) signal to temporally separate optical and molecular responses. The developed sensor is insensitive to baseline laser intensity imperfections and spectral interference from other species. Accurate measurements of methane in the presence of a representative interfering species, benzene, are performed by careful selection of the scan index (ratio of laser tuning range to spectral linewidth) and initial and final time of m-FID signal fitting. The minimum detection limit of the sensor is ~ 110 ppm which can be enhanced with an optical cavity. The proposed sensing strategy can be utilized to measure methane leaks in harsh environments and in the presence of interfering species in environment-monitoring applications.

## Introduction

Methane has important astrophysical applications due to its significant presence in many planetary systems^1^, and has been detected in the atmosphere of Saturn, Titan, Jupiter, Uranus, Mars, and Pluto^2^. Trace quantities of methane in human breath can be used as a biomarker for intestinal problems and colonic fermentation^3^.

Methane is the third most important greenhouse gas in the Earth’s atmosphere after water vapor and carbon dioxide^4^, and its concentration has been increasing steadily due to anthropogenic activities^5^. Anthropogenic methane emissions are almost double than those from natural sources^6^, so methane is a key target for reductions of greenhouse-gas inventories. The Intergovernmental Panel on Climate Change (IPCG) has asked policy makers to develop methods for measuring and limiting the emissions of global-warming gases^7^. Methane is a major contributor to climate change and its global warming potential is ~ 25 times larger compared to CO_2_^8^. Reducing methane emissions is essential as methane contributes to ~ 25% of the current warming^9^. Pursuing mitigation strategies urgently can dwindle the warming rate and help avoid 0.25 °C increase by 2050, and more than 0.5 °C by 2100^10^. A myriad of sensing technologies have been developed to mitigate methane emissions^11^.

Methane is the main constituent ($\sim$ 90%) of natural gas (NG). Accidental explosions in NG/air mixtures are very costly in terms of lives, materials, and mental health of people^12^. The Richmond Hill explosion in 2012 occurred due to massive leaks of methane, which accumulated in a partially enclosed area, which got ignited and led to a catastrophe^13^. Astrophysical explosions have been linked to deflagration-to-detonation transitions (DDT), which have been investigated in channels containing methane/air mixtures^12^.

Absorption spectroscopy provides quantitative, non-intrusive measurements in various systems^14,15^. Numerous laser absorption sensors have been developed for methane detection in gas sensing applications. These sensors have been developed in both the mid- and near infrared regions of the methane absorption spectrum. Mid-infrared laser sources are being increasingly employed as absorption strengths of most hydrocarbons are orders of magnitude higher in the mid-IR compared to the near IR region^16–18^. Direct absorption and photoacoustic techniques have been employed to develop atmospheric methane sensors using distributed feedback (DFB) diode lasers operating in the near IR region around 1.6 μm to access the 2v_3_ band of methane^19–22^. In addition, mid-IR methane sensors were reported using difference-frequency-generation (DFG) sources which emit near 3.3–3.4 μm to cover the v3 methane ro-vibrational band^23–27^. Recently, quantum cascade lasers (QCLs) enabled methane sensing near 8 μm targeting the ν_4_ band of methane^28–31^.

Difference frequency generation (DFG) systems, based on nonlinear optics, are quite complex and have low power. Quantum cascade lasers (QCLs) and interband cascade lasers (ICLs) are more compact, robust, and user-friendly, and have attracted high popularity for methane detection. However, previous sensing strategies were not designed to detect methane in the presence of strong absorption interference from other species.

Differential absorption (peak-minus-valley) takes advantage of narrow spectral features of target species to account for interference from broadly absorbing molecules^27^. Multidimensional linear regression can be applied along with scanned-wavelength absorption to split the absorbance spectrum into contributions from absorbing species^32^. However, these methods are subject to significant errors in the presence of fluctuations/imperfections in baseline laser intensity $\left( {I_{0} } \right)$ or the transmitted intensity $\left( {I_{t} } \right)$. In harsh environments, instability in laser intensity, shifting due to non-ideal transmission, the formation of etalons, and the presence of interfering species represent challenges to accurate absorption measurements, and can introduce significant errors when quantifying the target species.

Cepstral analysis was initially developed for audio signal processing^33^. This approach can be used convert the measured transmission spectrum to a time-domain modified free induction decay signal (m-FID). Here, much of the molecular response is temporally separated and becomes independent of the source intensity. Cepstral analysis can avoid errors in baseline laser intensity, which typically varies slowly in the optical domain and thus decays rapidly in the time domain. This method was demonstrated by Cole et al. to quantify methane in ethane bath gas over a wide tuning range (500 cm^−1^). Their technique was limited by a 90-min averaging time, and the necessity of informing the diagnostic about the presence of non-methane (i.e., ethane) components to calibrate the broadening coefficients of methane. Cepstral analysis has shown effectiveness in baseline-free sensing. However, interference from unknown absorbing species has not been investigated using this method, which is of importance in environmental monitoring applications.

Here, we report a novel, laser-based mid-IR sensor for interference-free measurements of methane. Cepstral analysis was applied on scanned-wavelength laser signals to design a sensor which is insensitive to baseline distortion or broadband interference from typical species in the atmosphere.

The following section reviews some existing baseline correction techniques and introduces the proposed m-FID method. We then conclude with an experimental test that demonstrates our approach for baseline-free and interference-free methane concentration measurements.

## Baseline correction techniques

Researchers have investigated multiple techniques to suppress errors caused by fluctuations in the baseline laser intensity, etalon effects, and interfering absorbance. In direct absorption spectroscopy (DAS), intensity variation is accounted for by polynomial/spline fitting^34^. While this approach is effective in a multitude of circumstances, it is susceptible to user bias and errors induced through the coupling between fitted polynomials and reference absorbance spectra. In the case of broadband absorbance spectra, access to non-absorbing spectral regions is minimal and thus artificial baseline generation is tricky.

Wavelength modulation spectroscopy (WMS) can mitigate the effect of broadband intensity variations^35,36^. This method has been effective in scanning the laser wavelength across absorption transitions while being modulated at a much higher frequency^37^. However, non-linear laser intensity response and/or etalon effects bewilder the ability of this method to account for variations in the background signal.

Cavity ring-down spectroscopy (CRDS) circumvents the need for baseline intensity^38^. Instead of measuring the attenuation of light, CRDS measures the decay time of transmitted signal rather than its magnitude^39^. CRDS provides highly sensitive measurements for trace detection of target species via an optical cavity formed with highly reflective mirrors. However, the optical alignment process is tedious and highly sensitive to mechanical fluctuations / vibrations.

Time-domain spectroscopy is a possible alternative to absorption spectroscopy, where it measures the free induction decay (FID) of molecules excited by pulsed radiation^40,41^. This technique shows promise as a baseline-free method since much of the molecular response is temporally separated from the laser excitation. However, gas property extraction using this approach requires exhaustive fitting to account for the intensity of the excitation pulse as it affects the magnitude of the FID signal^42^.

Bayesian statistics was recently employed to directly estimate the absorbance spectrum from the transmitted intensity data^43^. While this approach shows promising results, it was only validated using a limited set of water vapor simulated spectra. Additionally, Bayesian statistics inference is non-trivial and its algorithm adds significant processing complexity. Here, prior information of the shape of the baseline intensity is essential for absorbance estimation.

Cepstral analysis was first used with traditional absorbance spectrometers by Cole et al. in 2019 to analyze the transmitted signal independently of the baseline intensity by creating a modified form of the time-domain molecular free-induction decay (m-FID) signal^44^. Much of the m-FID signal is temporally separated from the laser source intensity, and gas properties can be retrieved by fitting this portion to a reference model. This eliminates the need to account/correct for the laser source intensity. Later, Goldenstein et al. developed an improved model by predicting the baseline intensity^45^. Validation of this technique was carried out by scanning the laser across two CO absorption transitions. Makowiecki et al. used this approach to scale reference absorption cross-sections to different pressures^46^. This was done by computationally adjusting the decay rate of the FID signal to account for collisional broadening of cross-sections. Recently, Li et al. optimized this method by careful selection of scan index and initial and final time of the fitting^47^. This method was demonstrated by targeting a CO_2_ transition near 4.2 µm. Cepstral analysis proved to be insensitive to errors in the baseline intensity which varies slowly in the optical domain and, therefore, decays rapidly in the time domain.

## Theoretical background

The fundamentals of absorption spectroscopy and modified free-induction-decay (m-FID) are briefly explained in “Absorption spectroscopy” and “Modified free-induction-decay (m-FID)” sections, respectively.

### Absorption spectroscopy

In absorption spectroscopy, a laser source emits a beam at frequency, $\nu$, and incident intensity, $I_{0}$, and the transmitted intensity, $I_{t}$, are collected after passing through an absorbing medium. The incident and transmitted laser intensities are related to the molecular absorbance, $\alpha$, through Beer–Lambert law^48^:

1 $$ I_{t} \left( \nu \right) = I_{0} \left( \nu \right)\exp \left[ { - \alpha \left( \nu \right)} \right] $$

2 $$ \alpha = \sum\limits_{j} {S_{j} (T)P\chi \phi_{j} \left( \nu \right)L} $$

where $S_{j} (T)$ is the temperature-dependent line-strength of the spectral transition $j$, $P$ is the total pressure of the gaseous mixture, $\chi$ is the mole fraction of the absorbing species, $\phi_{j} \left( \nu \right)$ is the frequency-dependent line-shape function of the transition $j$, and $L$ is the laser path-length through the absorbing medium. For a given absorption transition at known experimental conditions $\left( {\nu ,\,T,\,P,\,L} \right)$, the mole fraction $\chi$ can be inferred by fitting the measured absorbance to a simulated absorbance, as shown in “Fitting algorithm of the m-FID signal” section.

### Modified free-induction-decay (m-FID)

Free induction decay (FID) has been used in the processing of audio signals, and is determined by the inverse Fourier transform of the target signal. m-FID was introduced by Cole et al. in 2019^44^, where they derived the traditional time domain free induction decay of $I_{t} \left( \nu \right)$ through cepstral analysis. Forming the FID signal of the transmitted laser intensity, obtained from Eq. (1), results in a convolution of the incident intensity with the molecular response, as shown in Eq. (3):

3 $$ {\mathscr{F}}^{ - 1} \left[ {I_{t} \left( \nu \right)} \right] = {\mathscr{F}}^{ - 1} \left[ {I_{0} \left( \nu \right)} \right] * {\mathscr{F}}^{ - 1} \left\{ {\exp \left[ { - \alpha \left( \nu \right)} \right]} \right\} $$

If we modify Eq. (1) by taking its negative natural logarithm before applying the inverse Fourier transform, the additive relation formed in Eq. (4) will be conserved, as shown in Eq. (5):

4 $$ - \ln \left[ {I_{t} \left( \nu \right)} \right] = \alpha \left( \nu \right) - \ln \left[ {I_{0} \left( \nu \right)} \right] $$

5 $$ {\mathscr{F}}^{ - 1} \left\{ { - \ln \left[ {I_{t} \left( \nu \right)} \right]} \right\} = {\mathscr{F}}^{ - 1} \left[ {\alpha \left( \nu \right)} \right] - {\mathscr{F}}^{ - 1} \left\{ { - \ln \left[ {I_{0} \left( \nu \right)} \right]} \right\} $$

The LHS of Eq. (5) is the m-FID, which corresponds to the cepstrum of the time domain transmitted intensity signal. In the case of narrow absorbance features, the laser intensity will vary slower than the molecular response in the optical frequency domain, and the laser intensity will thus decay faster in the time domain. In contrast, the term corresponding to the molecular absorption response in the time domain of Eq. (5) periodically oscillates before decaying to zero after a relatively long time. This happens because all absorbing molecules are initially excited at nearly the same time, so they rotate in phase and emit radiation into the same mode as the laser source, causing the m-FID signal. Due to the differences in the rotational energy/speed of the excited molecules, they will soon rotate out of phase and no longer emit into the laser mode. However, additional bursts will be emitted after periodically re-phasing the molecules due to the quantized rotational rates, causing additional m-FID signals. This pattern will fade after some time due to the collisions^49^.

After the baseline intensity term in Eq. (5) decays, the unaffected portion of the m-FID signal can be least-squares fitted to a simulated absorbance signal of the target species to determine its properties. This is achieved through a Levenberg–Marquardt algorithm^50^ with careful selection of the initial and final time of the fitting.

## Sensor description

**Figure 1:**
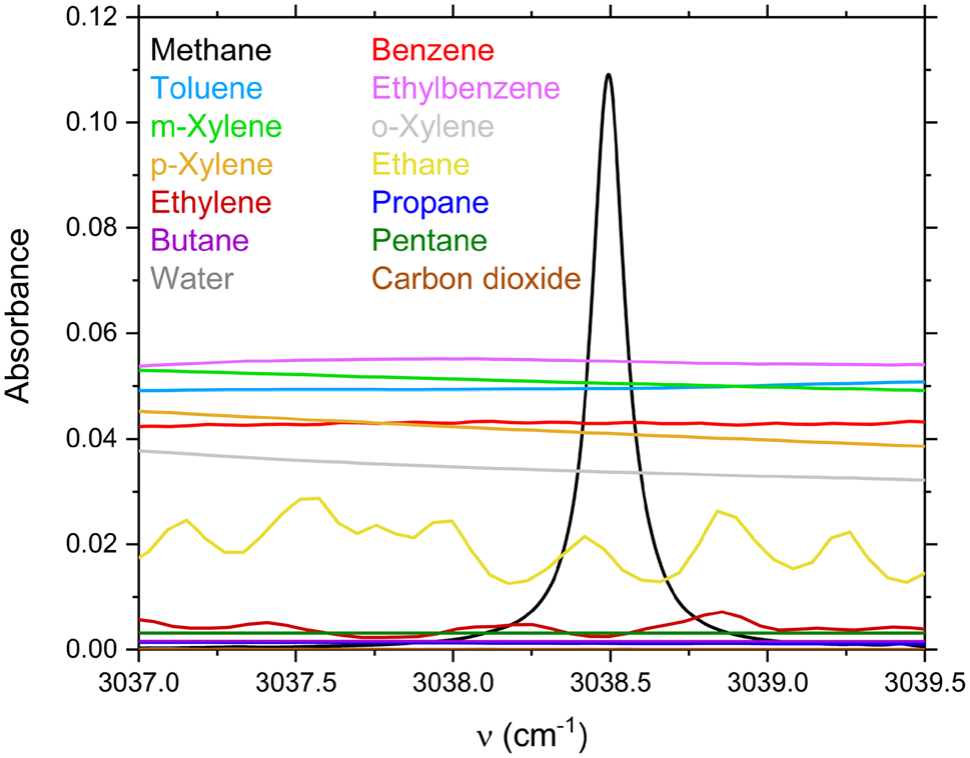
Absorbance spectra for methane, benzene, toluene, ethylbenzene, m-xylene, o-xylene, p-xylene, ethane, ethylene, propane, butane, pentane, water vapor, and carbon dioxide over 3037–3039.5 cm^-1^ at T = 298 K, P = 1 atm, L = 10 cm^51,52^.

Methane has strong absorption with dense transition lines in the 3–3.5 μm region^51^. Sensors operating near 3.3 μm that cover the ${\nu }_{3}$ band of methane have been reported to achieve higher sensitivity compared to sensors operating away from 3.3 μm^26^. Figure 1 shows the absorbance spectra of methane, benzene, toluene, ethylbenzene, m-xylene, o-xylene, p-xylene, ethane, ethylene, propane, butane, pentane, water vapor, and carbon dioxide in the range of 3037 – 3039.5 cm^-1^ at $T$ = 298 K, $P$ = 1 atm, $L$ = 10 cm^51,52^. These spectra were calculated for 1000 ppm concentration of all species except water vapor (2%). These species were chosen as the major constituents of the atmospheric gases and VOC emissions. It is obvious that ethylene, propane, butane, pentane, water vapor, and carbon dioxide have negligible absorbance in our target spectral range. While ethane spectrum appears to have some features, these ethane features are broad compared to methane features. In fact, the frequency domain gradient of the methane line is ~ 20 times larger than that of ethane, which means that the m-FID signal of ethane will decay much faster than that of methane in the time domain for the selected wavelength region. Also, the amount of ethane in air is typically much lower than that of methane, so its m-FID signal will be weaker. Therefore, for environmental monitoring applications, the main interfering species to methane at 3.3 μm are BTEX species (benzene, toluene, ethylbenzene, and xylenes). Due to the similar absorbance spectra of BTEX species^53^, benzene is chosen as a representative molecule for simplicity. In the case of methane and benzene absorption, Eq. (5) becomes:

6 $$ {\mathscr{F}}^{ - 1} \left\{ { - \ln \left[ {I_{t} } \right]} \right\} = {\mathscr{F}}^{ - 1} \left[ {\alpha_{Methane} } \right] + {\mathscr{F}}^{ - 1} \left[ {\alpha_{Benzene} } \right] - {\mathscr{F}}^{ - 1} \left\{ { - \ln \left[ {I_{0} } \right]} \right\} $$

Given the broad and slowly varying absorbance of benzene (see Fig. 1), its m-FID signal decays rapidly in the time domain, similar to the $I_{0}$ signal. Thus, selecting an initial fitting time large enough for the decay enables interference-free measurements of methane.

**Figure 2:**
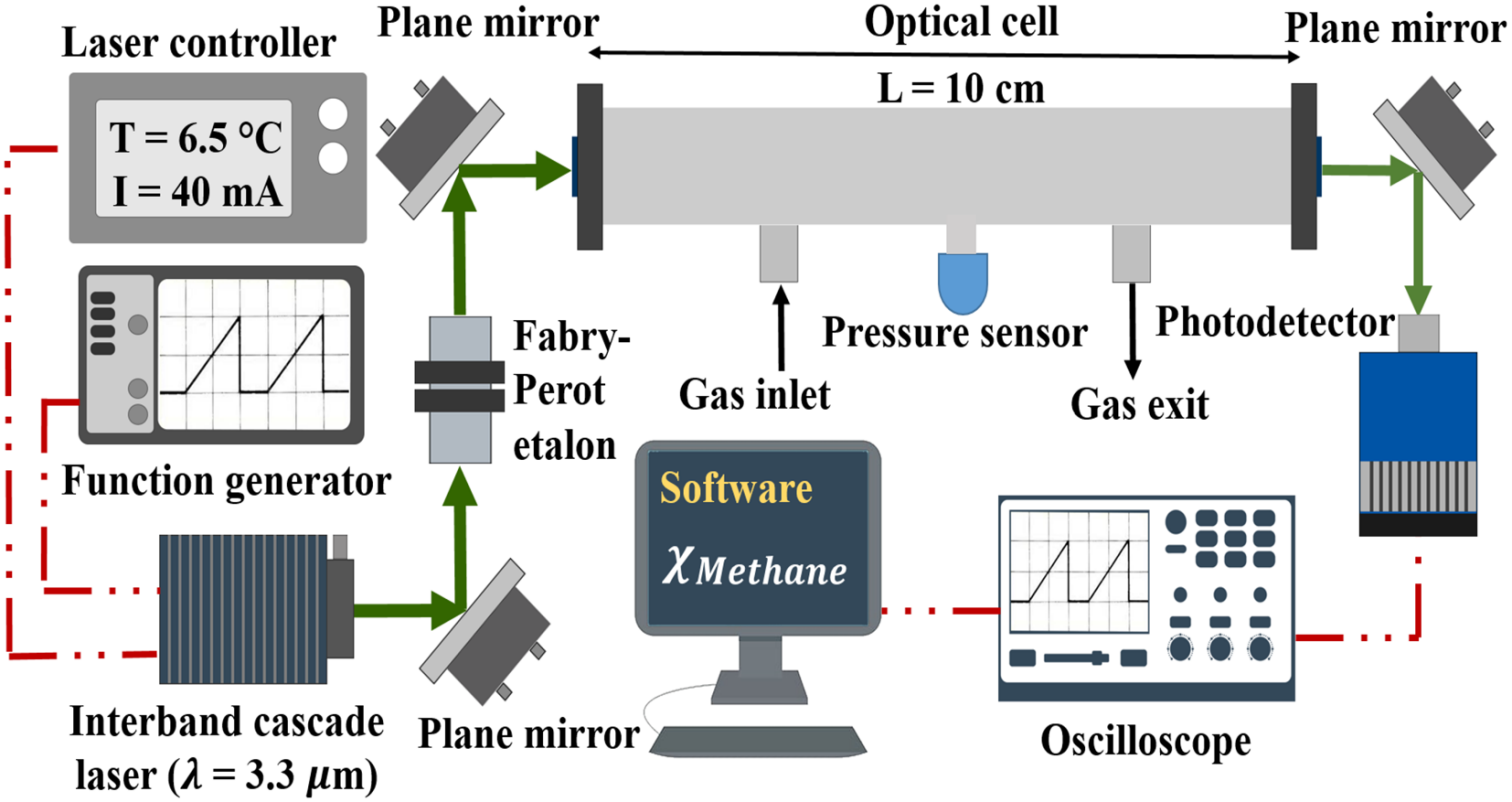
Optical schematic of the methane sensor.

The sensor uses a distributed feedback interband cascade laser (3290 nm DFB-ICL, Nanoplus) emitting near 3.3 µm with an output power ~ 1 mW^54^. Two ZnSe windows (Thorlabs, WG71050-E4) were mounted on a 10-cm gas sampling cell. The transmitted signal was collected with a DC-coupled, TE-cooled photodetector (1.5 MHz bandwidth, Vigo Systems, PVI-4TE-3.4–2 × 2). The laser wavelength was tuned over 3037–3039.5 cm^−1^ by a linear ramp of the laser injection current at 1 kHz scan rate, and a 7.62-cm germanium Fabry–Pérot etalon was used to convert the scan time to frequency (wavenumbers). Figure 2 shows the schematic of the sensor setup. All measurements were carried out at a static pressure of 1 atm.

Li et al. observed that for a small scan index (ratio of laser tuning range to spectral linewidth), most of the m-FID signal is concentrated in the early time period, i.e., it decays rapidly, which makes it difficult to separate the molecular response from the baseline intensity^47^. This means that higher scan index leads to longer decay time of the m-FID signal, which is desirable to minimize interference effects of the fast-decaying signals. Here, the tuning range of our laser was maximized to 2.5 cm^-1^ by scanning the laser injection current, within the laser’s allowable temperature and current limits, while the FWHM of the targeted methane line is ~ 0.25 cm^-1^, which resulted in a scan index of ~ 10.

## Fitting algorithm of the m-FID signal

**Figure 3:**
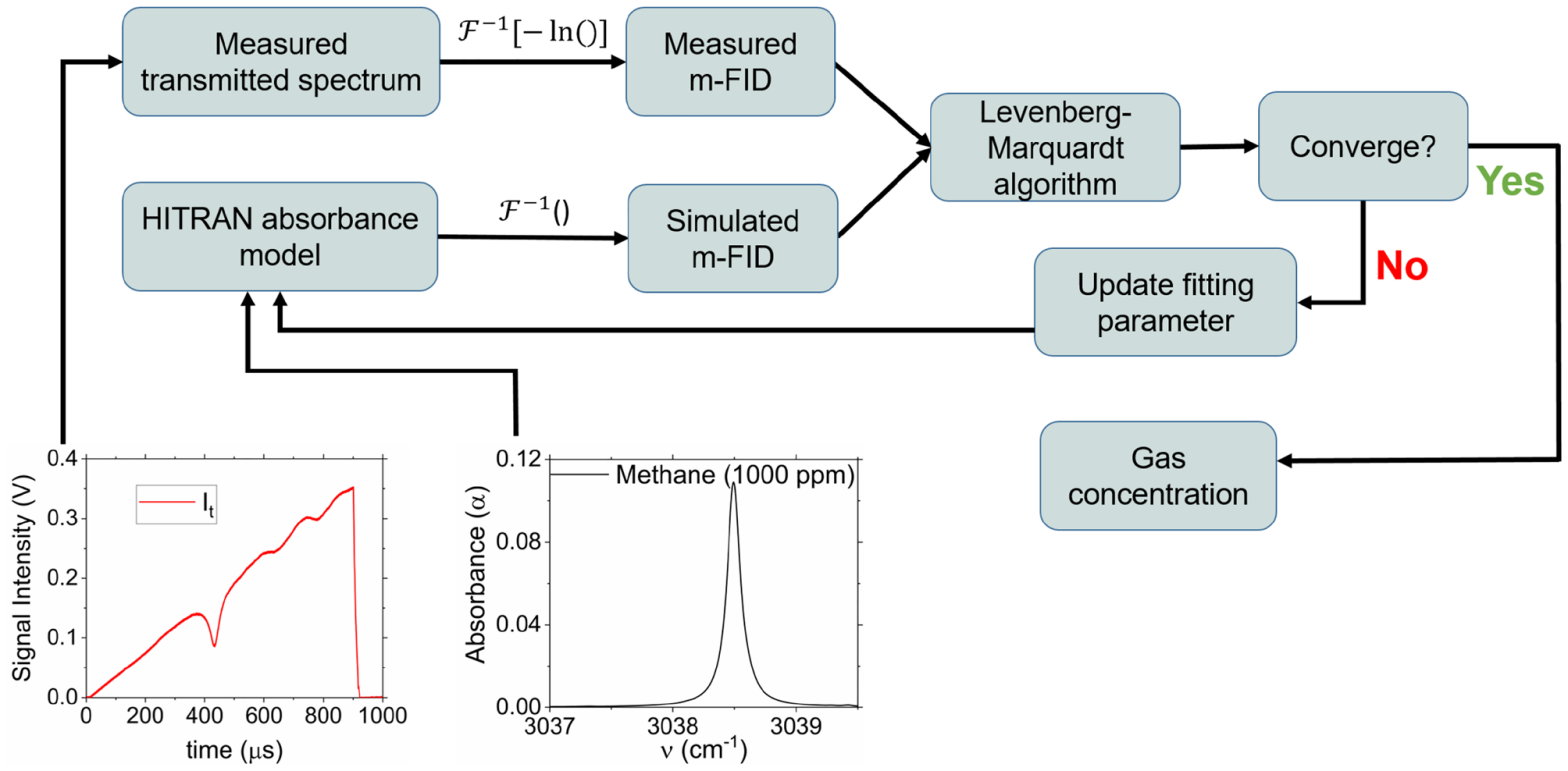
Flow chart illustrating the least-squares fitting of the m-FID signal to retrieve methane concentration.

This section describes our technique to retrieve methane concentration from the measured transmitted laser intensity using the m-FID signal. In cepstral analysis, the majority of molecular response is deconvolved from the influence of the baseline intensity. The uninfluenced portion of the m-FID signal can be least-squares-fit to a known model to obtain gas concentration. The fitting routine is illustrated in Fig. 3, where the inverse Fourier transform is calculated for the negative natural logarithm of the measured transmitted intensity and the simulated absorbance model at experimental conditions $\left( {\nu ,\,T,\,P,\,L,\,\chi } \right)$. The resulting cepstra are fed into the Levenberg–Marquardt fitting algorithm^50^. The fitting parameter is initially assumed to be 1, meaning that the target gas concentration in the mixture is equal to the reference concentration in the model, and the model keeps updating its fitting parameter until convergence. The final converged model yields the measured concentration of methane.

## Experimental results

### Reference validation

**Figure 4:**
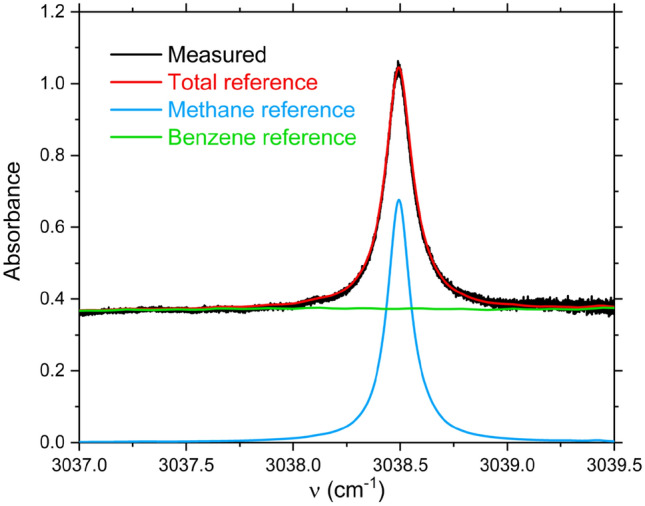
Composite measured and simulated absorbance spectra^51,52^ over 3037–3039.5 cm^−1^ at T = 298 K, P = 1 atm, L = 10 cm, χ_Methane_ = 6200 ppm, χ_Benzene_ = 8900 ppm.

Our m-FID approach was assessed by measuring the composition of methane/benzene mixtures in air. To validate our technique, a mixture containing 6200 ppm methane and 8900 ppm benzene in air was prepared manometrically. Mixture composition was verified by a traditional absorption experiment where etalon effects were removed through careful optical alignment and laser fluctuations were minimized by time averaging. Here, both the baseline and transmitted laser intensities were measured to calculate the composite absorbance shown in Fig. 4. Good agreement between measured and simulated absorbance confirms mixture composition and paves the way towards testing our approach in imperfect/realistic experimental conditions where etalon effects and intensity fluctuations are not mitigated.

### Laser intensity imperfections

**Figure 5:**
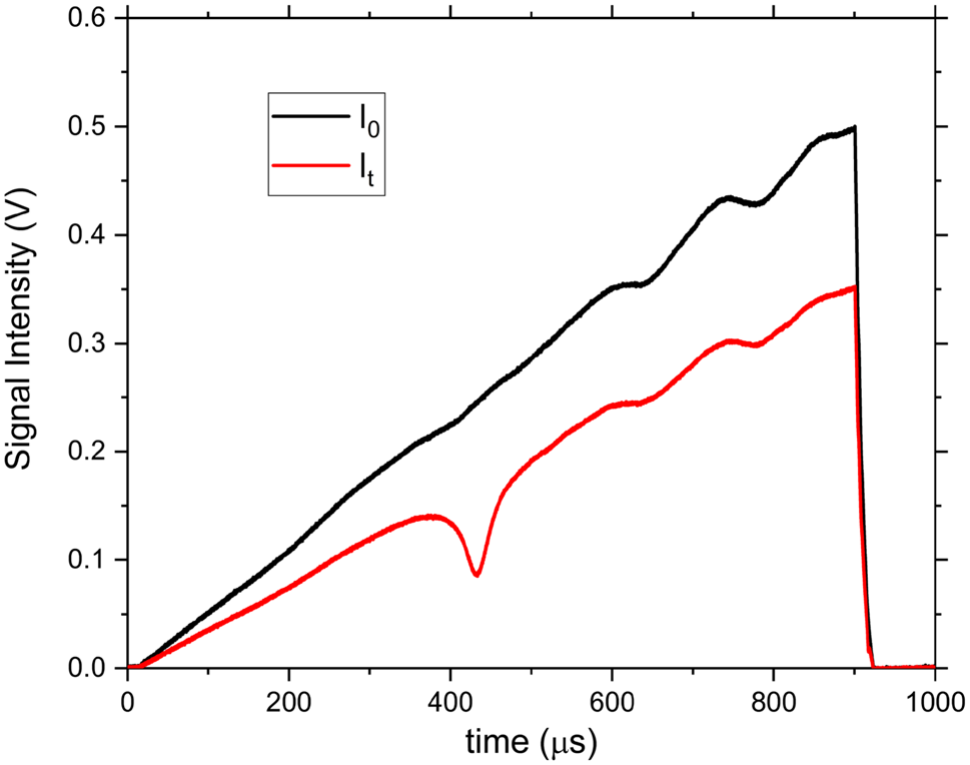
Intensities of the incident and transmitted signals through a mixture of 6200 ppm methane and 8900 ppm benzene in air.

Here, optical alignment was intentionally distorted to introduce imperfections to the laser intensity. Figure 5 shows the incident and transmitted laser intensities through the mixture of 6200 ppm methane and 8900 ppm benzene in air. Due to the broadband absorption of benzene and the absence of non-absorbing regions, as shown in Fig. 1, it becomes challenging to separate any laser fluctuations from the measured absorbance. In addition, etalon effects due to planar optics (e.g., windows and/or plane mirrors) add more complexity to the retrieval of gas concentration, so etalons were intentionally preserved here, as shown in Fig. 5, to test the power of m-FID method. Thus, the intensity attenuation can be due to a combination of molecular absorbance, baseline fluctuations, and etalons.

### m-FID signal fitting

**Figure 6:**
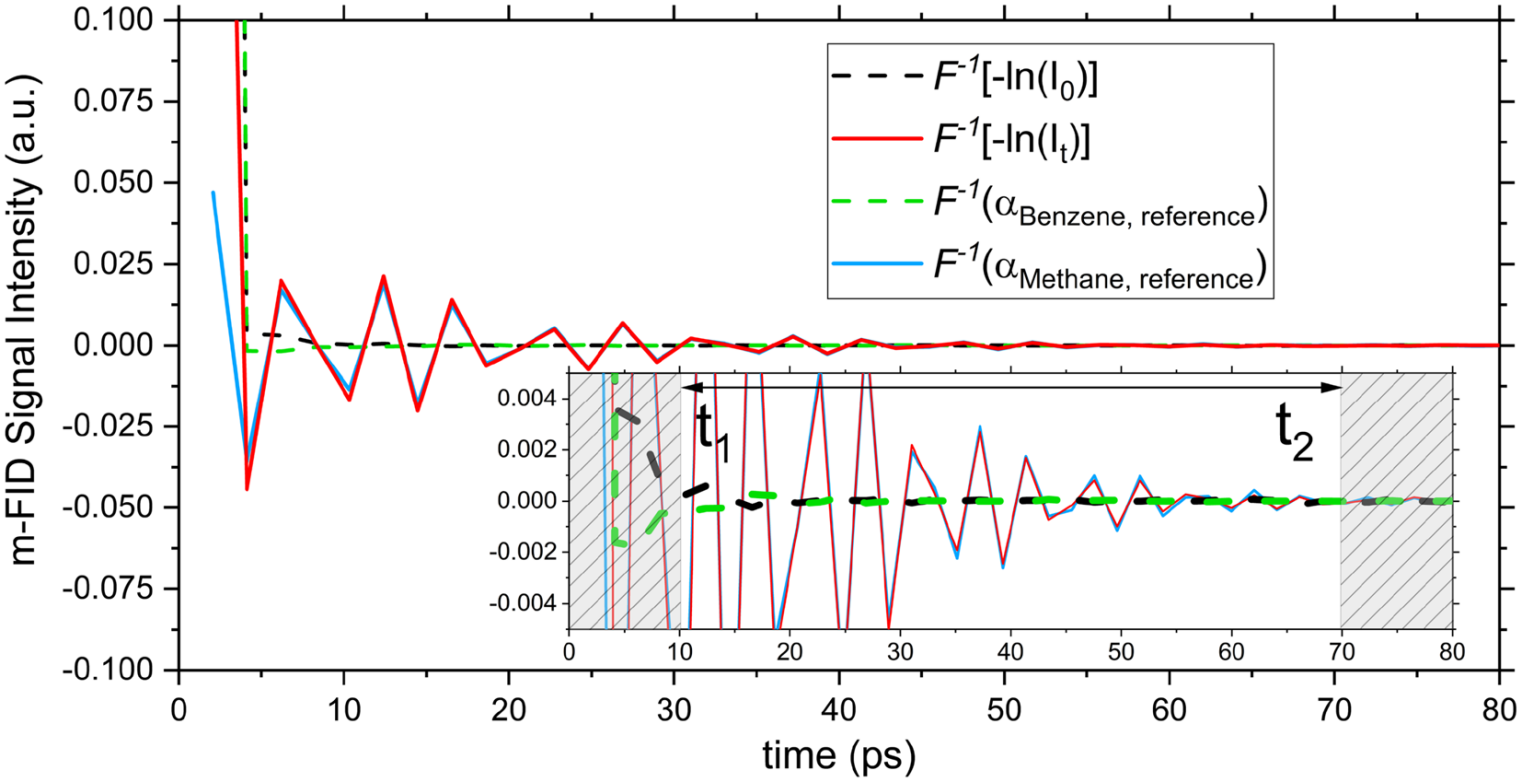
Time domain m-FID signals of −ln(I_0_), −ln(I_t_), α_Benzene_ and α_Methane_. The inset is a zoom-in view of the vertical axis to show the decay of the signals.

Inverse Fourier transform was applied to the incident and transmitted intensity signals, shown in Fig. 5, and to the simulated absorbance of methane and benzene shown in Fig. 1. The resulting time-domain m-FID signals are shown in Fig. 6, which constitute the components of Eq. (6). The terms corresponding to smooth signals in the frequency domain, i.e., $I_{0}$ and $\alpha_{Benzene}$, decay rapidly with time. After decay, the Levenberg–Marquardt algorithm was employed to least-squares fit the remaining m-FID signals corresponding to $I_{t}$ and $\alpha_{Methane}$ to infer methane concentration.

The fitting is done over a time window between initial time, $t_{1}$, and final time, $t_{2}$, according to the step function:

7 $$ F\left( T \right) = \left\{ {\begin{array}{*{20}l} 0 & {\forall \;t \in \;\;\left[ {0,\,t_{1} } \right[ \cup \left] {t_{2} ,\, + \infty } \right]} \\ 1 & {\forall \;t \in \;\;\left[ {t_{1} ,\,t_{2} } \right]} \\ \end{array} } \right. $$

The initial time controls how much of the early-time part of the m-FID signal of $I_{t}$ is ignored by the fitting algorithm. A small $t_{1}$ value retains the influence of the rapidly decaying signals, which introduces errors to the fitting. However, using a too large $t_{1}$ value results in the elimination of a significant part of the target (methane) absorbance response, which also adds errors to the fitting algorithm. Hence, $t_{1}$ was chosen so that the signals corresponding to $I_{0}$ and $\alpha_{Benzene}$ decay to within 0.1% of their initial values. On the other hand, $t_{2}$ should be sufficiently large to retain most of the m-FID signal, but should not exceed the value when the signal intensity becomes less than 0.1% of its initial value. The inset in Fig. 6 is zoomed in to the vertical axis to show the signals’ decay. Here, $t_{1}$ and $t_{2}$ were selected to be 10 ps and 70 ps, respectively.

### Sensor validation and minimum detection limit

**Figure 7:**
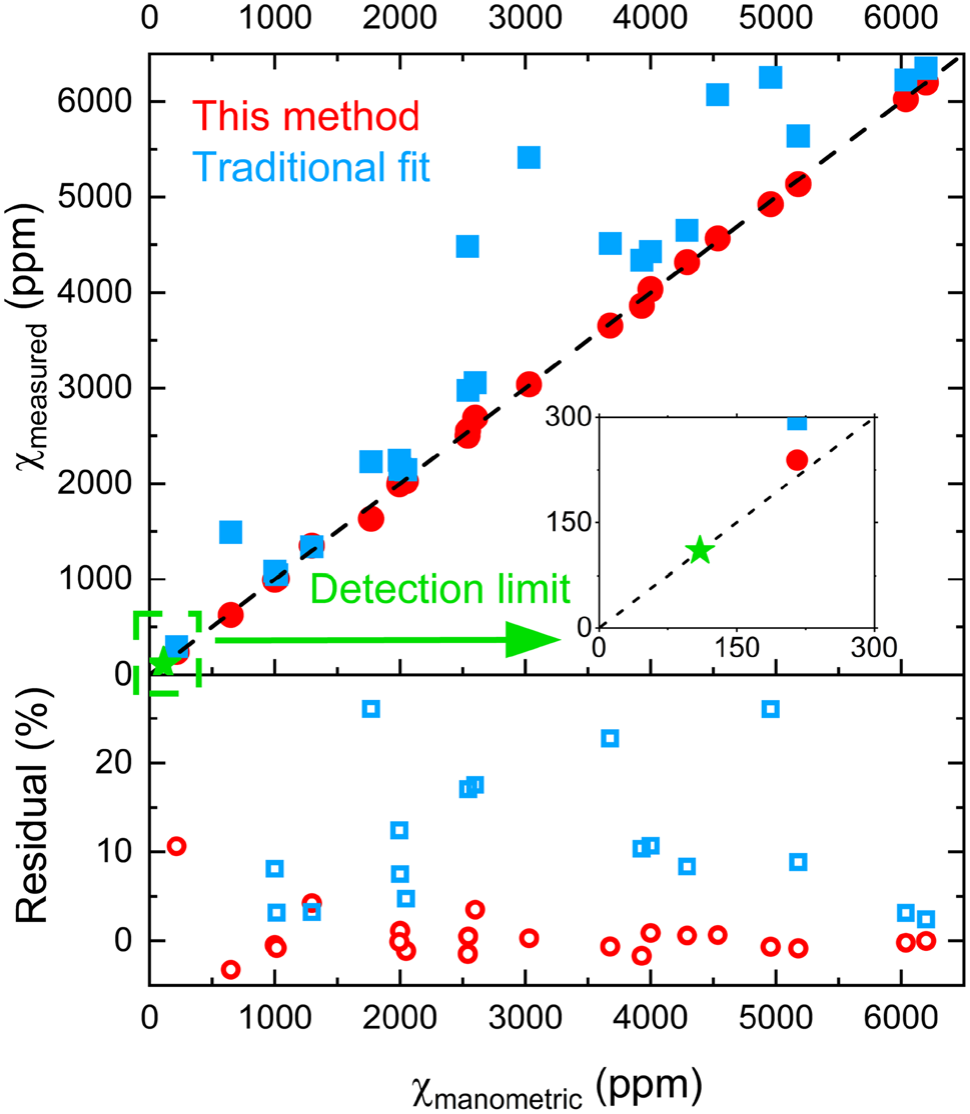
Comparison of measured and manometric methane mole fraction values. Minimum detection limit is 110 ppm at the experimental conditions.

Measurements were performed on various mixtures of methane and benzene in air. Methane concentration was varied over 216–6200 ppm, while that of benzene spanned 0–10,000 ppm. The results, based on m-FID approach (red circles), are plotted in Fig. 7 which shows remarkable agreement between the measured (via m-FID method) and manometric methane concentrations at $T$ = 298 K and $P$ = 1 atm. Figure 7 also shows the results obtained from a traditional least squares fitting of the measured absorbance to the reference methane absorbance (blue squares). The large discrepancy between the measured (via traditional method) and manometric mole fractions is due to the presence of benzene in the measured samples, which significantly contributes to the composite absorbance. Benzene interference is alleviated in the m-FID approach due to its rapid decay in the time domain. The bottom panel of Fig. 7 shows the residuals between the measured and manometric mole fractions of methane using m-FID and traditional approaches, with the former giving significantly superior results.

To quantify the minimum detection limit of the proposed sensor, an uncertainty analysis is carried out to estimate standard deviation of the fitting parameter by following the method of Adler et al.^55^. The reference absorbance spectrum is simulated at $N$ discrete frequencies, $\alpha_{R} \left( {\nu_{i} } \right)$, for given conditions $\left( {T,\,P,\,\chi } \right)$, and its corresponding m-FID signal is denoted by $A_{R} \left( {t_{i} } \right)$ at times $t_{i}$ in time domain. The measured transmitted intensity $I_{T} \left( {\nu_{i} } \right)$, is assumed to have a uniform noise of standard deviation $\sigma_{I}$ over the frequency domain. The corresponding m-FID signal in time domain is denoted by $M_{T} \left( {t_{i} } \right)$, and has a uniform time domain standard deviation $\sigma_{M}$. The fitting parameter $\zeta$ is used to retrieve the actual gas concentration $\left( {\chi_{measured} = \zeta \cdot \chi_{reference} } \right)$, and it is found by minimizing the differences between $\zeta \cdot A_{R} \left( {t_{i} } \right)$ and $M_{T} \left( {t_{i} } \right)$. The Levenberg–Marquardt optimization function in the time fitting window is given as:

8 $$ L(\zeta ) = \sum\limits_{i}^{N} {F\left( {t_{i} } \right) \cdot \left[ {\zeta \cdot A_{R} \left( {t_{i} } \right) - M_{T} (t_{i} )} \right]^{2} = \min } $$

9 $$ \frac{dL(\zeta )}{{d\zeta }} = \sum\limits_{i}^{N} {2F\left( {t_{i} } \right) \cdot \left[ {\zeta \cdot A_{R} \left( {t_{i} } \right) - M_{T} (t_{i} )} \right] \cdot \left[ {A_{R} \left( {t_{i} } \right)} \right]} = 0 $$

Simplifying Eq. (9) gives:

10 $$ \zeta = \frac{{\sum\nolimits_{i}^{N} {F\left( {t_{i} } \right) \cdot } A_{R} \left( {t_{i} } \right) \cdot M_{T} (t_{i} )}}{{\sum\nolimits_{i}^{N} {F\left( {t_{i} } \right) \cdot } A_{R}^{2} \left( {t_{i} } \right)}} $$

11 $$ \sigma_{\zeta }^{2} = \sum\limits_{i}^{N} {\left[ {\frac{\partial \zeta }{{\partial M_{T} (t_{i} )}}} \right]^{2} \cdot \sigma_{M}^{2} } = \sum\limits_{i}^{N} {\left[ {\frac{{F(t_{i} ) \cdot A_{R} (t_{i} )}}{{\sum\nolimits_{i}^{N} {F(t_{i} ) \cdot } A_{R}^{2} (t_{i} )}}} \right]^{2} } \cdot \sigma_{M}^{2} $$

Thus, an explicit form of the standard deviation of the fitting parameter is given as:

12 $$ \sigma_{\zeta } = \frac{{\left[ {\sum\nolimits_{i}^{N} {F^{2} (t_{i} ) \cdot } A_{R}^{2} (t_{i} )} \right]^{\frac{1}{2}} }}{{\sum\nolimits_{i}^{N} {F(t_{i} ) \cdot } A_{R}^{2} (t_{i} )}} \cdot \sigma_{M} $$

Here, the step function is chosen to be unity in Eq. (7). Therefore, Eq. (12) reduces to:

13 $$ \sigma_{\zeta } = \left[ {\sum\limits_{i}^{N} {A_{R}^{2} (t_{i} )} } \right]^{{ - \frac{1}{2}}} \cdot \sigma_{M} $$

Knowing the standard deviation of the fitting parameter $\left( {\sigma_{\zeta } } \right)$ and the simulated concentration ($\chi$ = 1000 ppm), the theoretical minimum detection limit (MDL) of the sensor ($T$ = 23 °C, $P$ = 1 atm, $L$ = 10 cm) is estimated to be MDL = $\sigma_{\zeta } \cdot \chi$≈ 50 ppm. The input noise power of the photodetector, $P_{in}$, is calculated by dividing the input noise current by the detector responsivity^56^. The noise equivalent power, $NEP$, is then calculated by:

14 $$ NEP = \frac{{P_{in} }}{{\sqrt {BW} }} $$

where $BW$ is the bandwidth of the photodetector (20 MHZ in this case). The specific detectivity of the photodetector, D*, is derived by relating the active area, $A$, to $NEP$, as follows:

15 $$ D* = \frac{\sqrt A }{{NEP}} $$

The specific detectivity was calculated to be $7\times {10}^{11}\mathrm{cm}\sqrt{\mathrm{Hz}}/\mathrm{W}$. This controls the noise level in the measured laser intensity signal ($I_{t}$), which translates into a noise equivalent concentration, $NEC$, of 110 ppm. Experimental detection limit is determined from the noise level of the m-FID signals of $I_{0}$ and $I_{t}$ given in Fig. 6. This was scaled with respect to the simulated concentration ($\chi$ = 1000 ppm) to result in an experimental detection limit of 90 ppm. Considering these estimates of the MDL, we take the largest of the three values (110 ppm) to be the conservative MDL of our sensor.

## Concluding remarks

A laser sensor based on absorption spectroscopy has been developed for interference-free and baseline-free measurements of methane concentration. The technique generates an m-FID signal using cepstral analysis, which enables the separation of methane absorbance from benzene absorbance and baseline laser intensity. The laser was tuned over 2.5 cm^−1^ range at a scan index of ~ 10, and the transmitted laser intensity was least-squares fitted to a simulated methane absorbance signal in the time domain to infer methane concentration. The fitting window was restricted between 10 and 70 ps to avoid the influence of interference and baseline intensity. The sensor has a minimum detection limit of ~ 110 ppm at T = 23 °C and P = 1 atm, and can be utilized to measure methane concentration at ambient conditions. Laser path-length can be increased using a longer optical cell, multi-pass cell, or an optical cavity to decrease MDL by orders of magnitude. For example, replacing the optical windows with 99.97% reflectivity mirrors would result in a cavity gain of 3332, and thus would reduce MDL to ~ 50 ppb. In harsh environments, the technique greatly reduces the need for baseline intensity correction which can introduce significant error, and the technique also adequately accounts for broadband absorption interference.

## Data Availability

Data underlying the results presented in this paper are not publicly available at this time but may be obtained from the authors upon reasonable request.
